# *N*,*N*′-Bis(pyridin-3-ylmeth­yl)ethanedi­amide monohydrate: crystal structure, Hirshfeld surface analysis and computational study

**DOI:** 10.1107/S2056989019016153

**Published:** 2020-01-01

**Authors:** Sang Loon Tan, Edward R. T. Tiekink

**Affiliations:** a Research Centre for Crystalline Materials, School of Science and Technology, Sunway University, 47500 Bandar Sunway, Selangor Darul Ehsan, Malaysia

**Keywords:** crystal structure, di­amide, hydrogen bonding, Hirshfeld surface analysis, computational chemistry

## Abstract

The mol­ecular structure of the title bis-pyridyl substituted di­amide hydrate, C_14_H_14_N_4_O_2_·H_2_O, features a central C_2_N_2_O_2_ residue (r.m.s. deviation = 0.0205 Å) linked at each end to 3-pyridyl rings through methyl­ene groups. The pyridyl rings lie to the same side of the plane, *i.e.* have a *syn*-periplanar relationship, and form dihedral angles of 59.71 (6) and 68.42 (6)° with the central plane. An almost orthogonal relationship between the pyridyl rings is indicated by the dihedral angle between them [87.86 (5)°]. Owing to an *anti* disposition between the carbonyl-O atoms in the core, two intra­molecular amide-N—H⋯O(carbon­yl) hydrogen bonds are formed, each closing an *S*(5) loop. Supra­molecular tapes are formed in the crystal *via* amide-N—H⋯O(carbon­yl) hydrogen bonds and ten-membered {⋯HNC_2_O}_2_ synthons. Two symmetry-related tapes are linked by a helical chain of hydrogen-bonded water mol­ecules *via* water-O—H⋯N(pyrid­yl) hydrogen bonds. The resulting aggregate is parallel to the *b*-axis direction. Links between these, *via* methyl­ene-C—H⋯O(water) and methyl­ene-C—H⋯π(pyrid­yl) inter­actions, give rise to a layer parallel to (10); the layers stack without directional inter­actions between them. The analysis of the Hirshfeld surfaces point to the importance of the specified hydrogen-bonding inter­actions, and to the significant influence of the water mol­ecule of crystallization upon the mol­ecular packing. The analysis also indicates the contribution of methyl­ene-C—H⋯O(carbon­yl) and pyridyl-C—H⋯C(carbon­yl) contacts to the stability of the inter-layer region. The calculated inter­action energies are consistent with importance of significant electrostatic attractions in the crystal.

## Abstract 2

The organic mol­ecule in the title bis-pyridyl-substituted di­amide hydrate has a U-shape as the 3-pyridyl rings lie to the same side of the central plane. In the crystal, two supra­molecular tapes, each sustained by amide-N—H⋯O(carbon­yl) hydrogen bonds and ten-membered {⋯HNC_2_O}_2_ synthons, are connected by a helical chain of hydrogen-bonded water mol­ecules.

## Chemical context   

Having both amide and pyridyl functionality, bis­(pyridin-*n*-ylmeth­yl)ethanedi­amide mol­ecules of the general formula *n*-NC_5_H_4_CH_2_N(H)C(=O)C(=O)CH_2_C_5_H_4_N-*n*, for *n* = 2, 3 and 4, hereafter *^n^L*H_2_, are attractive co-crystal coformers *via* conventional hydrogen bonding. In the same way, complexation to metals may also be envisaged. It is therefore not surprising that there is now a wealth of structural information for these mol­ecules occurring in co-crystals, salts and metal complexes, as has been reviewed recently (Tiekink, 2017 ▸). Complementing hydrogen-bonding inter­actions, the *^n^L*H_2_ mol­ecules, for *n* = 3 (Hursthouse *et al.*, 2003 ▸; Goroff *et al.*, 2005 ▸; Jin *et al.*, 2013 ▸) and *n* = 4 (Goroff *et al.*, 2005 ▸; Wilhelm *et al.*, 2008 ▸; Tan & Tiekink, 2019*c* ▸), are well-known to form N⋯I halogen-bonding inter­actions and, indeed, some of the earliest studies were at the forefront of pioneering systematic investigations of halogen bonding. It was during the course of on-going studies into co-crystal formation (Tan, Halcovitch *et al.*, 2019 ▸; Tan & Tiekink, 2019*a* ▸,*b* ▸,*c* ▸) and complexation to zinc(II) 1,1-di­thiol­ates (Arman *et al.*, 2018 ▸; Tiekink, 2018 ▸; Tan, Chun *et al.*, 2019 ▸), that the title compound, ^3^ *L*H_2_·H_2_O, (I), was isolated. Herein, the crystal and mol­ecular structures of (I) are described along with a detailed analysis of the mol­ecular packing by means of an analysis of the calculated Hirshfeld surfaces, two-dimensional fingerprint plots and the calculation of energies of inter­action.
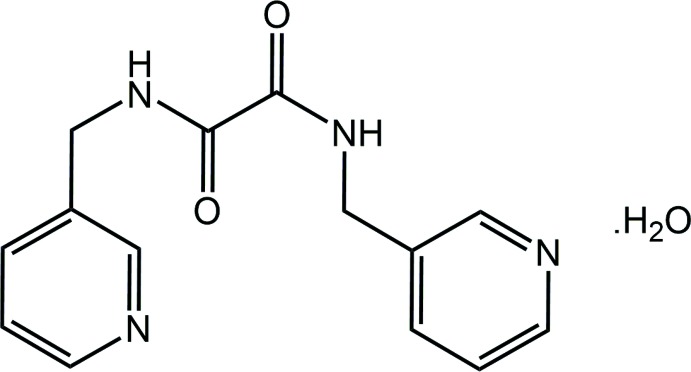


## Structural commentary   

**Figure 1:**
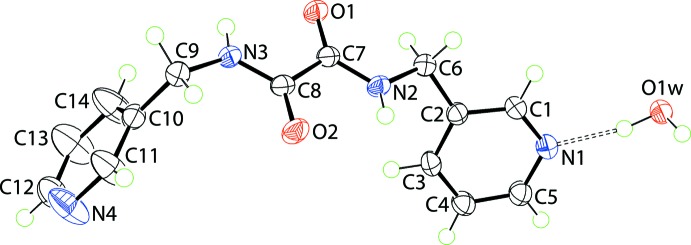
The mol­ecular structure of the constituents of (I) showing the atom-labelling scheme and displacement ellipsoids at the 70% probability level. The water-O—H⋯N(pyrid­yl) hydrogen bond is indicated by the dashed line.

The mol­ecular structures of the two constituents comprising the crystallographic asymmetric unit of (I) are shown in Fig. 1 ▸. The ^3^ *L*H_2_ mol­ecule lacks crystallographic symmetry and comprises a central C_2_N_2_O_2_ residue connected at either side to two 3-pyridyl residues *via* methyl­ene links. The six atoms of the central residue are almost co-planar as seen in their r.m.s. deviation of 0.0205 Å: the maximum deviations above and below the plane are 0.0291 (9) Å for N3 and 0.0321 (11) Å for C8. The N1- and N3-pyridyl rings form dihedral angles of 59.71 (6) and 68.42 (6)°, respectively, with the central plane and lie to the same side of the plane, having a *syn*-periplanar relationship. The dihedral angle formed between the pyridyl rings is 87.86 (5)°, indicating an almost edge-to-face relationship. The carbonyl-O atoms have an *anti* disposition enabling the formation of intra­molecular amide-N—H⋯O(carbon­yl) hydrogen bonds that close *S*(5) loops, Table 1 ▸.

## Supra­molecular features   

**Figure 2:**
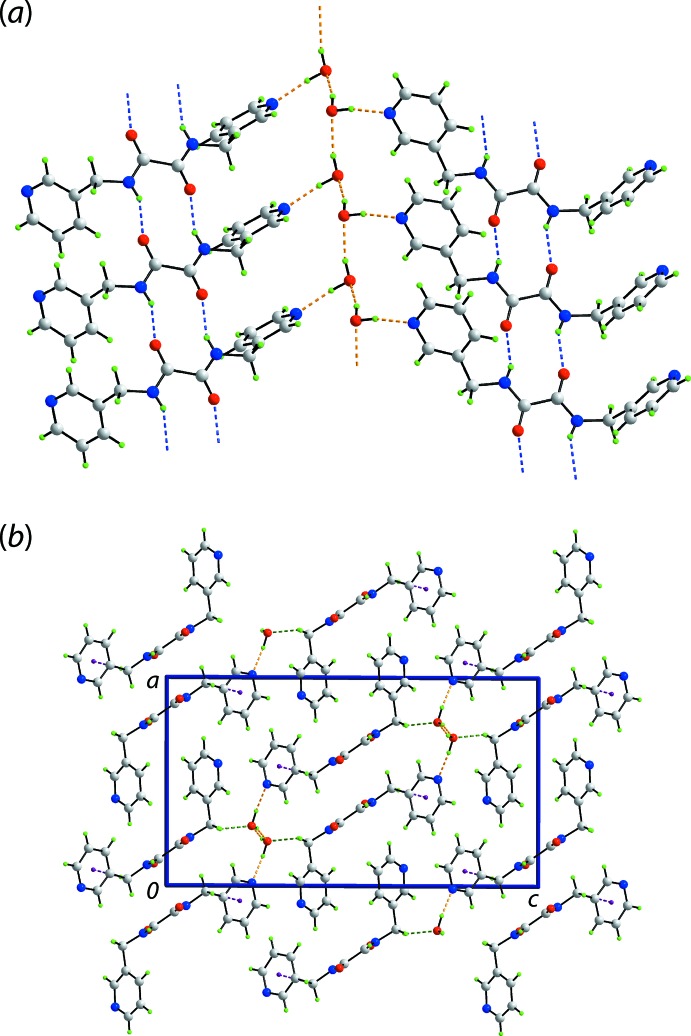
Mol­ecular packing in the crystal of (I): (*a*) one-dimensional chain whereby tapes sustained by amide-N—H⋯O(carbon­yl) hydrogen bonds and ten-membered {⋯HNC_2_O}_2_ synthons are connected, *via* water-O—H⋯N(pyrid­yl) hydrogen bonds, by helical chains of hydrogen-bonded water mol­ecules sustained by water-O—H⋯O(water) hydrogen bonds and (*b*) a view of the unit-cell contents in projection down the *b* axis, highlighting the stacking of layers. The amide-N—H⋯O(carbon­yl) hydrogen bonds are shown as blue dashed lines and hydrogen bonds involving the water mol­ecules, by orange dashed lines. The C—H⋯O and C—H⋯π inter­actions are shown as green and purple dashed lines, respectively.

Significant conventional hydrogen bonding is noted in the crystal of (I) with the geometric parameters characterizing these included in Table 1 ▸. The most striking feature of the supra­molecular association is the formation of tapes *via* amide-N—H⋯O(carbon­yl) hydrogen bonds leading to a sequence of inter-connected ten-membered {⋯HNC_2_O}_2_ synthons. Two such tapes are connected by hydrogen bonds provided by the water mol­ecule of crystallization. Thus, alternating water mol­ecules in helical chains of hydrogen-bonded water mol­ecules, being aligned along the *b*-axis direction and propagated by 2_1_ symmetry, connect to ^3^ *L*H_2_ *via* water-O—H⋯N(pyrid­yl) hydrogen bonds to form the one-dimensional aggregate shown in Fig. 2 ▸(*a*). The presence of methyl­ene-C—H⋯O(water) and methyl­ene-C—H⋯π(pyrid­yl) contacts stabilizes a layer lying parallel to (10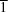). The layers stack without directional inter­actions between them, Fig. 2 ▸(*b*).

## Hirshfeld surface analysis   

**Figure 3:**
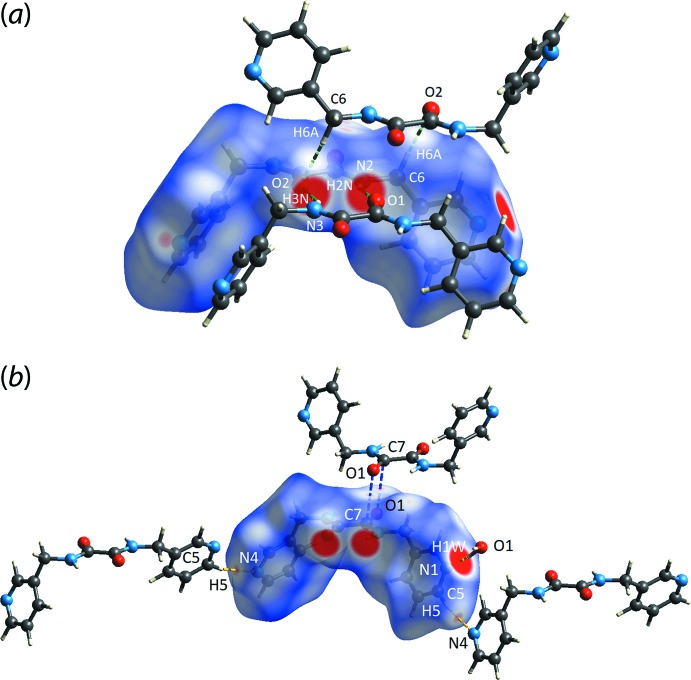
The *d*_norm_ mapping of the Hirshfeld surface for ^3^ *L*H_2_ in (I) within the range of −0.3259 to 1.0656 arbitrary units, showing the red spots for (*a*) N2—H2*N*⋯O1 (intense, connected by green dashed line), N3—H3*N*⋯O2 (intense, green dashed line) and C6—H6*A*⋯O2 (diminutive, green dashed line) inter­actions, (*b*) O1*W*—H1*W*⋯N1 (intense, yellow dashed line), C5—H5⋯N4 (moderately intense, yellow dashed line) and C7⋯O1 (diminutive, blue dashed line) inter­actions.

The calculations of the Hirshfeld surfaces and two-dimensional fingerprint plots were performed on the crystallographic asymmetric unit shown in Fig. 1 ▸, using *Crystal Explorer 17* (Turner *et al.*, 2017 ▸) and based on the procedures as described previously (Tan, Jotani *et al.*, 2019 ▸). The analysis identified a number of red spots on the *d*_norm_ surface of ^3^ *L*H_2_ with varying degrees of intensity indicating the presence of inter­actions with contact distances shorter than the sum of the respective van der Waals radii (Spackman & Jayatilaka, 2009 ▸). Referring to the images of Fig. 3 ▸, the most intense red spots stem from the amide-N—H⋯O(carbon­yl) and water-O—H⋯N(pyrid­yl) hydrogen bonds, Table 1 ▸. Some additional contacts are detected through the Hirshfeld surface analysis for C1—H1⋯O1*W*, C5–H5⋯N4, C12—H12⋯C7, C6–H6*A*⋯O2 and C7⋯O1 inter­actions with the red spots ranging from moderately to weakly intense. The data in Table 2 ▸ provide a succinct summary of inter­atomic contacts revealed in the above analysis; the O2⋯H6*A* and C7⋯H12 contacts occur in the inter-layer region.

**Figure 4:**
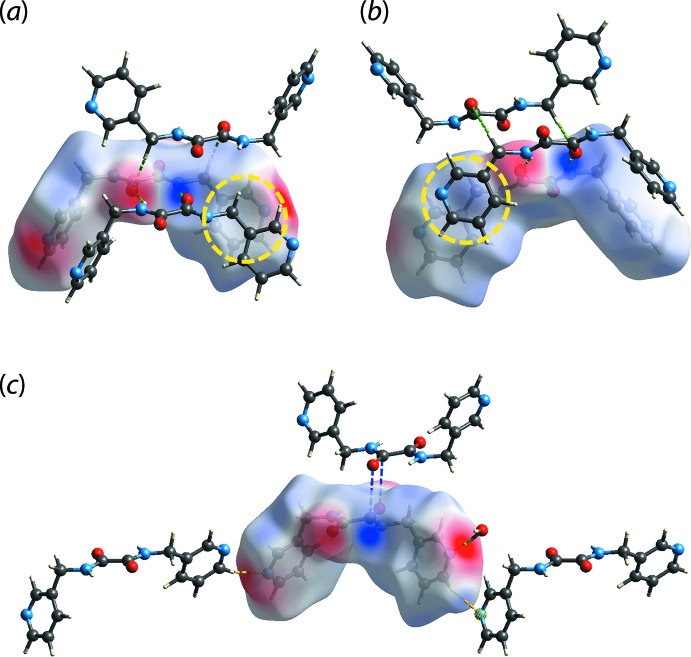
The electrostatic potential mapped onto the Hirshfeld surface within the isosurface value of −0.0964 to 0.1012 atomic units for ^3^ *L*H_2_ in (I), showing the charge complementarity for (*a*) C6—H6*A*⋯O2 (green dashed lines), (*b*) N2—H2*N*⋯O1 and N3—H3*N*⋯O2 (green dashed lines) and (*c*) C5—H5⋯N4 (yellow dashed line), O1*W*—H1*W*⋯N1 (yellow dashed line) and C7⋯O1 (blue dashed lines) inter­actions. The yellow circles in (*a*) and (*b*) highlight the dispersive nature of the methyl­ene-C—H⋯π(pyrid­yl) inter­action with no charge complementarity.

To verify the nature of the aforementioned inter­actions, the ^3^ *L*H_2_ mol­ecule in (I) was subjected to electrostatic potential mapping. The results show that almost all of the inter­actions identified through the *d*_norm_ mapping are electrostatic in nature as can be seen from the distinctive blue (electropositive) and red (electronegative) regions on the surface, albeit with varying intensity, Fig. 4 ▸. A notable exception is found for the methyl­ene-C—H⋯π(pyrid­yl) inter­action which is manifested in the pale regions in Fig. 4 ▸(*a*) and (*b*). This indicates no charge complementarity consistent with the inter­action beings mainly dispersive in nature.

**Figure 5:**
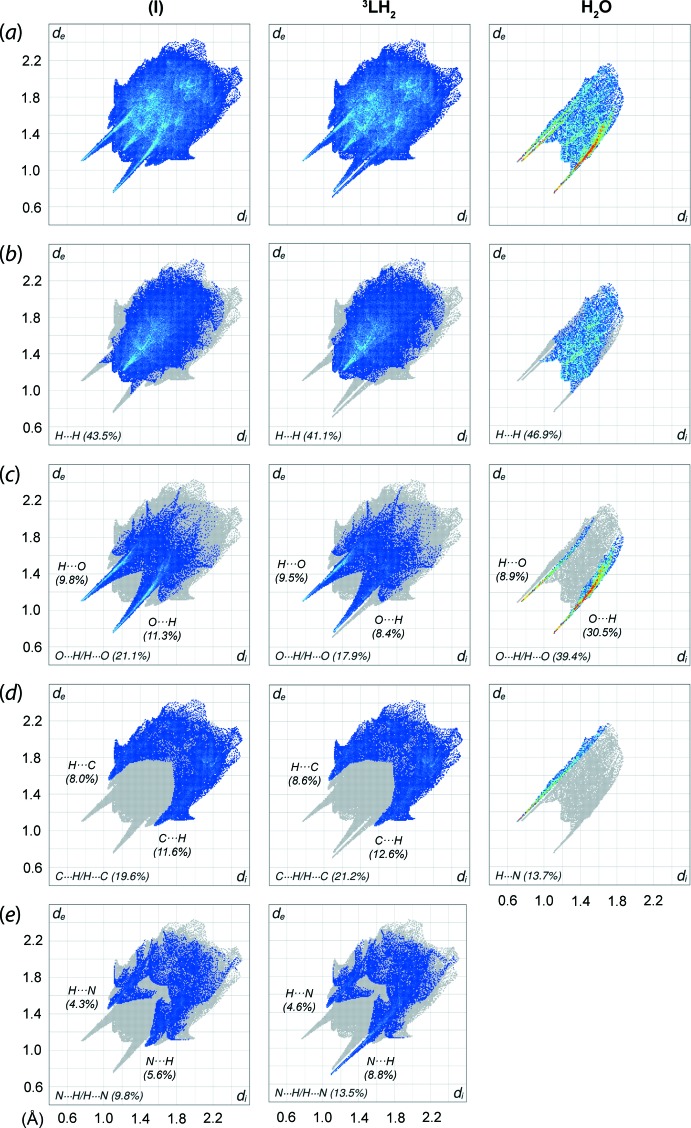
(*a*) The overall two-dimensional fingerprint plots for (I) and for the individual ^3^ *L*H_2_ and water mol­ecules, and those delineated into (*b*) H⋯H, (*c*) H⋯O/O⋯H, (*d*) H⋯C/C⋯H and (*e*) H⋯N/N⋯H contacts. The percentage contributions to the surfaces are indicated therein.

The qu­anti­fication of the close contacts to the Hirshfeld surface was performed through the analysis of the two-dimensional fingerprint plots for (I) as well as for the individual mol­ecular components. As shown in Fig. 5 ▸(*a*), the overall fingerprint plot of (I) exhibits a bug-like profile with a pair of symmetric spikes. This is in contrast to the asymmetric profile of ^3^ *L*H_2_, with splitting of the spike in the inter­nal region due to the formation of the O—H⋯N hydrogen bond, Fig. 5 ▸(*e*), suggesting a prominent role played by the water mol­ecule in influencing the overall contacts in (I). The observation is very different to that of the benzene solvate of ^4^ *L*H_2_ in which the overall surface contacts for ^4^ *L*H_2_ are not very much influenced by the benzene mol­ecule as demonstrated by the similar profiles for the solvate and individual ^4^ *L*H_2_ mol­ecule (Tan, Halcovitch *et al.*, 2019 ▸). The decomposition of the overall profile of (I) shows that the most significant contacts are primarily H⋯H contacts (43.5%), followed by O⋯H/H⋯O (21.1%), C⋯H/H⋯C (19.6%) and N⋯H/H⋯N (9.8%) contacts, with all of these inter­actions having *d*_i_ + *d*_e_ distances less than the respective sums of van der Waals radii (vdW), *i.e.* H⋯H ∼2.26 Å [Σ(vdW) = 2.40 Å], O⋯H/H⋯O ∼1.88 Å [Σ(vdW) = 2.72 Å], C⋯H/H⋯C ∼2.62 Å [Σ(vdW) = 2.90 Å] and N⋯H/H⋯N ∼2.50 Å [Σ(vdW) = 2.75 Å].

As for the individual ^3^LH_2_ mol­ecule, the dominance of these contacts follows the order H⋯H (41.1%; *d*_i_ + *d*_e_ 2.33 Å), C⋯H/H⋯C (21.2%; *d*_i_ + *d*_e_ 2.60 Å), O⋯H/H⋯O (17.9%; *d*_i_ + *d*_e_ 1.88 Å) and N⋯H/H⋯N (13.5%; *d*_i_ + *d*_e_ 1.80 Å). While the aforementioned inter­actions are almost evenly distributed between the inter­nal and external contacts for (I), some contacts for ^3^ *L*H_2_ are found to either to be inclined towards the inter­nal or external contact region compared with (I), such as that displayed by (inter­nal)-O⋯H-(external) (8.4%) *versus* (inter­nal)-H⋯O-(external) (9.5%) and (inter­nal)-N⋯H-(external) (8.8%) *versus* (inter­nal)-H⋯N-(external) (4.6%), respectively, Fig. 5 ▸(*c*)–(*e*).

The hydrate mol­ecule exhibits a completely different fingerprint profile, which is dominated by three major contacts, namely H⋯H (46.9%; *d*_i_ + *d*_e_ 2.26 Å), O⋯H/H⋯O (39.4%; *d*_i_ + *d*_e_ 1.88 Å) and H⋯N (13.7%; *d*_i_ + *d*_e_ 1.80 Å). In particular, the second most dominant contacts are found to be heavily inclined toward (inter­nal)-O⋯H-(external) (30.5%) as compared to (inter­nal)-H⋯O-(external) (8.9%), presumably due to relatively large contact surface area.

## Computational chemistry   

All associations between mol­ecules in (I), as described in *Hirshfeld surface analysis*, were subjected to the calculation of the inter­action energy using *Crystal Explorer 17* (Turner *et al.*, 2017 ▸) based on the method described previously (Tan, Jotani *et al.*, 2019 ▸) to evaluate the strength of each inter­action, Table 3 ▸. Among those close contacts, the (^3^ *L*H_2_)_2_ dimer connected by a ten-membered {⋯HNC_2_O}_2_ synthon has the greatest *E*_int_ energy of −73.0 kJ mol^−1^ which is comparable in energy to the classical eight-membered {⋯HOCO}_2_ synthon (Tan & Tiekink, 2019*a* ▸). Perhaps unexpectedly, the C12–H12⋯C7 contact which also sustains a pair of ^3^ *L*H_2_ mol­ecules constitutes the second strongest inter­action with *E*_int_ = −32.7 kJ mol^−1^, and this is followed by the C6—H6*A*⋯O2 (−32.0 kJ mol^−1^), O1*W*—H1*W*⋯N1 (−28.6 kJ mol^−1^), O1*W*—H2*W*⋯O1*W* (−26.2 kJ mol^−1^), C7⋯O1 (−20.7 kJ mol^−1^), C5—H5⋯N4 (−13.0 kJ mol^−1^) and C1—H1⋯O1*W* (−10.5 kJ mol^−1^) inter­actions. As expected, the N2—H2*N*⋯O1, N3—H3*N*⋯O2, O1*W*—H1*W*⋯N1 and O1*W*—H2*W*⋯O1*W* inter­actions are associated with distinct electropositive and electronegative sites and therefore, are mainly governed by electrostatic forces, while the rest of the close contacts are dispersive in nature. The relatively stable nature of the C12—H12⋯C7 and C6—H6*A*⋯O2 inter­actions as compared to the O1*W*—H1*W*⋯N1 and O1*W*—H2*W*⋯O1*W* inter­actions could be due to the presence of low repulsion energies in the former as compared to the latter.

**Figure 6:**
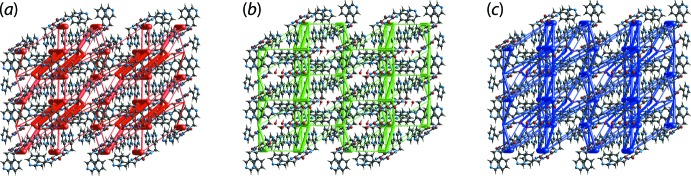
Perspective views of the energy framework of (I), showing the (*a*) electrostatic force, (*b*) dispersion force and (*c*) total energy diagram. The cylindrical radius is proportional to the relative strength of the corresponding energies and they were adjusted to the same scale factor of 100 with a cut-off value of 8 kJ mol^−1^ within 2 × 1 × 2 unit cells.

The crystal of (I) is mainly sustained by electrostatic forces owing to the strong N2—H2*N*⋯O1/ N3—H3*N*⋯O2, O1*W*—H1*W*⋯N1 and O1*W*—H2*W*⋯O1*W* hydrogen bonding leading to a barricade-like electrostatic energy framework parallel to (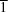
01), as shown in Fig. 6 ▸(*a*). This is further stabilized by the dispersion forces arising from other supporting inter­actions which result in another barricade-like dispersion energy framework parallel to (100), Fig. 6 ▸(*b*). The overall energy framework for (I) is shown in Fig. 6 ▸(*c*).

A comparison of the distribution of contacts on the Hirshfeld surfaces between the ^3^ *L*H_2_ mol­ecule in (I) and in its two polymorphic forms, *i.e*. Form I and Form II (Jotani *et al.*, 2016 ▸), with latter having two independent mol­ecules, was performed. This analysis returned the data shown in Table 4 ▸ and indicates that ^3^ *L*H_2_ in (I) is relatively closer to Form I as compared to the independent mol­ecules comprising Form II.

**Figure 7:**
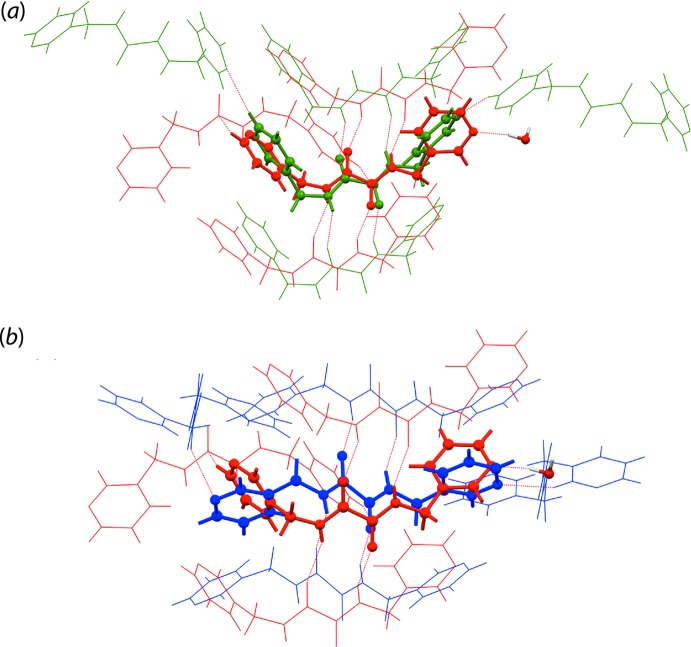
A comparison of the mol­ecular packing of ^3^ *L*H_2_: (*a*) (I) (red) and Form I (green) and (*b*) (I) (red) and Form II (blue), showing the differences in terms of mol­ecular connectivity of ^3^ *L*H_2_ with r.m.s. deviations of 0.895 and 1.581 Å, respectively.

This conclusion is consistent with the analysis of the packing similarity in which a comparison of (I) and Form I exhibits an r.m.s. deviation of 0.895 Å while a comparison with Form II exhibits an r.m.s. deviation of 1.581 Å, despite only one out of 20 mol­ecules displaying some similarity with the reference ^3^ *L*H_2_ mol­ecule in (I), Fig. 7 ▸. The packing analysis was performed using *Mercury* (Macrae *et al.*, 2006 ▸), with the analysis criteria being set that only mol­ecules within the 20% tolerance for both distances and angles were included in the calculation while mol­ecules with a variation >20% were discarded, and that mol­ecular inversions were allowed during calculation. It is therefore also apparent through this analysis that the water mol­ecules in (I) play a crucial role in influencing the packing of ^3^ *L*H_2_ in (I).

## Database survey   

The ^3^ *L*H_2_ mol­ecule has been characterized in two polymorphs (Jotani *et al.*, 2016 ▸) and in a number of (neutral) co-crystals. A characteristic of these structures is a long central C—C bond and conformational flexibility in terms of the relative disposition of the 3-pyridyl substituents with respect to the central C_2_N_2_O_2_ chromophore (Tiekink, 2017 ▸). Indeed, the relatively long length of the central C—C bonds often attracts a level C alert in *PLATON* (Spek, 2009 ▸). Of the data included in Table 5 ▸ [for the chemical diagrams of (II) and (III), see Scheme 2], the shorter of the C—C bonds is 1.515 (3) Å, found in the co-crystal of ^3^ *L*H_2_ with HO_2_CCH_2_N(H)C(=O)N(H)CH_2_CO_2_H (Nguyen *et al.*, 2001 ▸) and the longest bond of 1.550 (17) Å is found in the co-crystal of ^3^ *L*H_2_ with (III) (Jin *et al.*, 2013 ▸). In terms of conformational flexibility, the two polymorphs of ^3^ *L*H_2_ highlight this characteristic of these mol­ecules (Jotani *et al.*, 2016 ▸). In Form I, the pyridyl rings lie to the same side of the central C_2_N_2_O_2_ and therefore, have a *syn*-periplanar relationship, or, more simply, a U-shape. In Form II, comprising two independent mol­ecules, each is disposed about a centre of inversion so the relationship is *anti*-periplanar, or S-shaped. DFT calculations revealed that the difference in energy between the two conformations is less than 1 kcal^−1^ (Jotani *et al.*, 2016 ▸). Despite this result, most of the ^3^ *L*H_2_ mol­ecules are centrosymmetric, S-shaped. For the U-shaped mol­ecules, the dihedral angles between the central plane and pyridyl rings range from 59.71 (6) to 84.61 (9)°. The comparable range for the S-shaped mol­ecules, for which both dihedral angles are identical from symmetry, is 64.2 (3) to 84.79 (18)°.
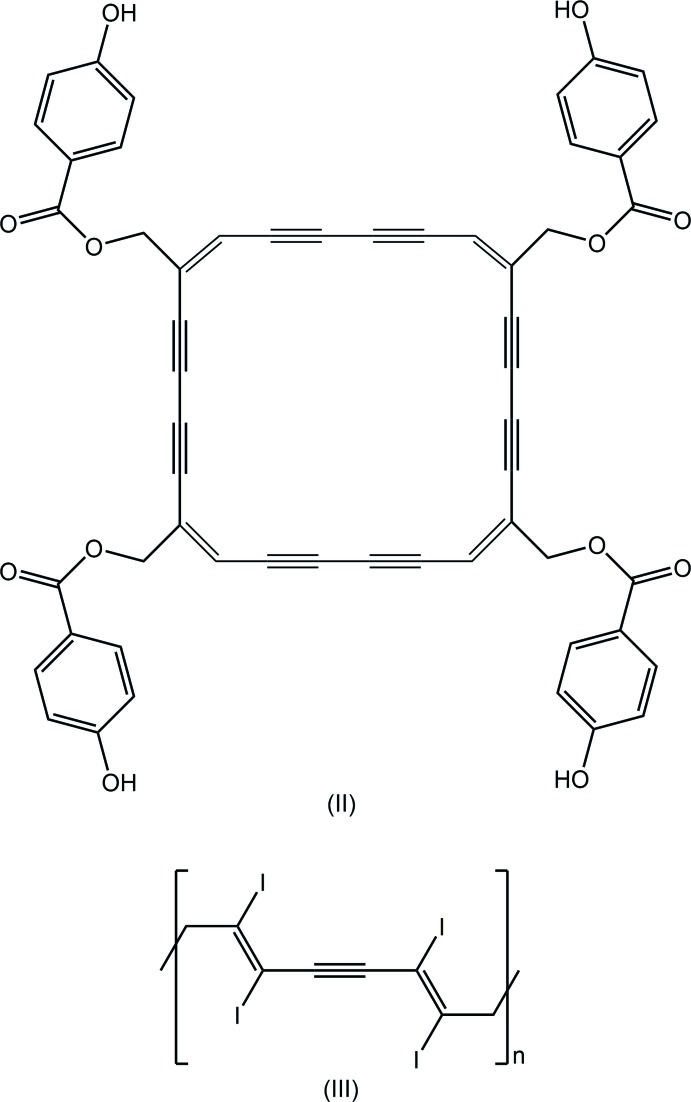


## Synthesis and crystallization   

The precursor, *N*,*N*′-bis­(pyridin-3-ylmeth­yl)oxalamide, was prepared according to the literature (Schauer *et al.*, 1997 ▸). Crystallization of the precursor in a DMF (1 ml) and ethanol (1 ml) mixture resulted in the isolation of the title hydrate, (I); m.p.: 409.4–410.7 K. IR (cm^−1^): 3578 ν(O—H), 3321 ν(N—H), 3141–2804 ν(C—H), 1687–1649 ν(C=O), 1524–1482 ν(C=C), 1426 ν(C—N), 710 ν(C=C).

## Refinement   

Crystal data, data collection and structure refinement details are summarized in Table 6 ▸. The carbon-bound H atoms were placed in calculated positions (C—H = 0.95–0.99 Å) and were included in the refinement in the riding-model approximation, with *U*_iso_(H) set to 1.2–1.5*U*_eq_(C). The oxygen- and nitro­gen-bound H atoms were located in a difference-Fourier map and refined with O—H = 0.84±0.01 Å and N—H = 0.88±0.01 Å, respectively, and with *U*_iso_(H) set to 1.5*U*_eq_(O) or 1.2*U*_eq_(N). Owing to poor agreement, one reflection, *i.e*. (551), was omitted from the final cycles of refinement.

## Supplementary Material

Crystal structure: contains datablock(s) I, global. DOI: 10.1107/S2056989019016153/hb7869sup1.cif

Structure factors: contains datablock(s) I. DOI: 10.1107/S2056989019016153/hb7869Isup2.hkl

Supporting information file. DOI: 10.1107/S2056989019016153/hb7869Isup3.cml

CCDC reference: 1969282

Additional supporting information:  crystallographic information; 3D view; checkCIF report

## Figures and Tables

**Table 1:** Hydrogen-bond geometry (Å, °)

*D*—H⋯*A*	*D*—H	H⋯*A*	*D*⋯*A*	*D*—H⋯*A*
N2—H2*N*⋯O2	0.85 (2)	2.36 (2)	2.7279 (18)	107.0 (16)
N3—H3*N*⋯O1	0.86 (2)	2.299 (19)	2.6924 (18)	108.0 (15)
O1*W*—H1*W*⋯N1	0.95 (2)	1.86 (2)	2.7958 (18)	169 (2)
O1*W*—H2*W*⋯O1*W* ^i^	0.88 (2)	1.97 (2)	2.8364 (15)	166 (2)
N2—H2*N*⋯O1^ii^	0.85 (2)	2.03 (2)	2.8227 (18)	155.2 (18)
N3—H3*N*⋯O2^iii^	0.86 (2)	2.02 (2)	2.8022 (18)	151.6 (17)
C9—H9*A*⋯O1*W* ^iv^	0.99	2.45	3.3772 (19)	156
C6—H6*B*⋯*Cg*1^iii^	0.99	2.74	3.7043 (16)	166

Symmetry codes: (i) 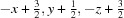; (ii) 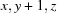; (iii) 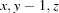; (iv) 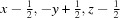.

**Table 2:** Summary of short inter­atomic contacts (Å) in (I)*^*a*^*

Contact	Distance	Symmetry operation
O2⋯H3*N*	1.89	*x*, 1 + *y*, *z*
O1⋯H2*N*	1.89	*x*, −1 + *y*, *z*
O2⋯H6*A*	2.57	1 − *x*, 1 − *y*, 1 − *z*
N4⋯H5	2.52	− 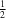 + *x*, 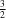 − *y*, − 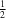 + *z*
C7⋯H12	2.64	−*x*, −*y*, 1 − *z*
O1*W*⋯H1	2.55	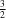 − *x*, 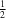 + *y*, 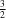 − *z*
C7⋯O1	3.16	1 − *x*, − *y*, 1 − *z*
N1⋯H1*W*	1.83	*x*, *y*, *z*

Notes: (*a*) The inter­atomic distances were calculated in *Crystal Explorer 17* (Turner *et al.*, 2017 ▸) whereby the *X*—H bond lengths are adjusted to their neutron values.

**Table 3:** Summary of inter­action energies (kJ mol^−1^) calculated for (I)

Contact	*E*_ele_	*E*_pol_	*E*_dis_	*E*_rep_	*E*_tot_
N2—H*2N*⋯O1^i^ +					
N3—H3*N*⋯O2^i^	−68.5	−15.0	−49.2	86.4	−73.0
C12—H12⋯C7^ii^	−6.7	−2.0	−46.1	26.0	−32.7
C6—H6*A*⋯O2^iii^	−12.9	−2.9	−28.2	13.5	−32.0
O1*W*—H*1W*⋯N1^iv^	−51.9	−11.2	−6.5	65.1	−28.6
O1*W*—H2*W*⋯O1*W* ^v^	−36.9	−7.1	−3.5	34.3	−26.2
C7⋯O1^vi^	−2.3	−3.0	−31.4	18.4	−20.7
C5—H5⋯N4^vii^	−9.4	−2.0	−8.1	8.7	−13.0
C1—H1⋯O1*W* ^viii^	−8.1	−1.3	−3.9	3.9	−10.5

Symmetry operations: (i) *x*, 1 + *y*, *z*; (ii) −*x*, −*y*, 1 − *z*; (iii) 1 − *x*, 1 − *y*, 1 − *z*; (iv) *x*, *y*, *z*; (v) 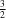 − *x*, 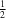 + *y*, 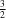 − *z*; (vi) 1 − *x*, − *y*, 1 − *z*; (vii) 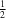 + *x*, 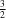 − *y*, 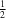 + *z*; (viii) 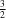 − *x*, −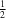 + *y*, 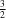 − *z*.

**Table 4:** A comparison of the distribution of contacts (%) to the calculated Hirshfeld surfaces for (I) and for Forms I and II (Jotani *et al.*, 2016 ▸)

Contact	(I)	Form I	Form IIa	Form IIb
H⋯H	41.1	44.1	35.8	36.9
C⋯H/H⋯C	21.2	16.7	31.4	22.4
O⋯H/H⋯O	17.9	15.7	14.2	19.6
N⋯H/H⋯N	13.5	16.7	18.0	19.5
C⋯O/O⋯C	2.3	2.1	0.1	0.1
Other	3.9	4.7	0.5	1.5

**Table 5:** Geometric data, *i.e.* central C—C bond lengths (Å) and dihedral angles (°), for ^3^ *L*H_2_ in its polymorphs, solvates and (neutral) co-crystals; see Scheme 2 for the chemical diagrams of (II) and (III)

Compound	Symmetry	Conformation	C—C	C_2_N_2_O_2_/(3-py)	(3-py)/(3-py)	REFCODE	Reference
Polymorphs							
Form I	–	U	1.544 (4)	74.98 (10), 84.61 (9)	88.40 (7)	OWOHAL	Jotani *et al.* (2016 ▸)
Form II*^*a*^*	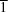	S	1.5383 (16)	77.29 (4)	0	OWOHAL01	Jotani *et al.* (2016 ▸)
	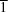	S	1.5460 (16)	75.93 (3)	0		
Solvate							
(I)	–	U	1.541 (2)	59.71 (6), 68.42 (6)	87.86 (5)	–	This work
Co-crystals of ^3^ *L*H_2_ with							
HO_2_CCH_2_N(H)C(=O)N(H)CH_2_CO_2_H	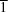	S	1.515 (3)	81.41 (7)	0	CAJQEK	Nguyen *et al.* (2001 ▸)
HO_2_CCH_2_N(H)C(=O)C(=O)N(H)CH_2_CO_2_H	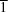	S	1.532 (19)	64.2 (3)	0	CAJQAG	Nguyen *et al.* (2001 ▸)
2-NH_2_C_6_H_4_CO_2_H	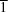	S	1.543 (2)	74.64 (4), 74.64 (4)	0	DIDZAT	Arman *et al.* (2012 ▸)
(II)	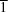	S	1.533 (3)	79.50 (6)	0	EMACIG	Suzuki *et al.* (2016 ▸)
C_6_F_4_I_2_	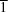	S	1.544 (4)	70.72 (9)	0	IPOSIP	Hursthouse *et al.* (2003 ▸)
2-HO_2_CC_6_H_4_SSC_6_H_4_CO_2_-2	–	U	1.543 (3)	61.22 (5), 69.43 (5)	72.12 (8)	KUZSOO	Arman *et al.* (2010 ▸)
4-NO_2_C_6_H_4_CO_2_H	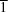	S	1.530 (2)	78.20 (4)	0	PAGFIP	Syed *et al.* (2016 ▸)
(III)	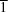	S	1.550 (17)	80.5 (4)	0	REWVUM	Jin *et al.* (2013 ▸)
I—C≡C—C≡C—I	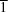	S	1.542 (10)	76.6 (2)	0	WANNOP	Goroff *et al.* (2005 ▸)
I—C≡C—C≡C—C≡C—I	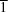	S	1.548 (11)	84.7 (2)	0	WANPIL	Goroff *et al.* (2005 ▸)
Br—C≡C—C≡C—Br	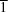	S	1.530 (9)	84.79 (18)	0	WUQQUW	Jin *et al.* (2015 ▸)

**Table 6:** Experimental details

Crystal data	
Chemical formula	C_14_H_14_N_4_O_2_·H_2_O
*M*_r_	288.31
Crystal system, space group	Monoclinic, *P*2_1_/*n*
Temperature (K)	100
*a*, *b*, *c* (Å)	12.4784 (4), 5.0247 (1), 22.2410 (6)
β (°)	90.170 (3)
*V* (Å^3^)	1394.51 (6)
*Z*	4
Radiation type	Cu *K*α
μ (mm^−1^)	0.82
Crystal size (mm)	0.09 × 0.07 × 0.03

Data collection	
Diffractometer	XtaLAB Synergy Dualflex AtlasS2
Absorption correction	Gaussian (*CrysAlis PRO*; Rigaku OD, 2018 ▸)
*T*_min_, *T*_max_	0.921, 1.000
No. of measured, independent and observed [*I* > 2σ(*I*)] reflections	16961, 2871, 2441
*R*_int_	0.053
(sin θ/λ)_max_ (Å^−1^)	0.631

Refinement	
*R*[*F* ^2^ > 2σ(*F* ^2^)], *wR*(*F* ^2^), *S*	0.043, 0.116, 1.04
No. of reflections	2871
No. of parameters	202
H-atom treatment	H atoms treated by a mixture of independent and constrained refinement
Δρ_max_, Δρ_min_ (e Å^−3^)	0.30, −0.24

Computer programs: *CrysAlis PRO* (Rigaku OD, 2018 ▸), *SHELXS* (Sheldrick, 2015*a* ▸), *SHELXL2017* (Sheldrick, 2015*b* ▸), *ORTEP-3 for Windows* (Farrugia, 2012 ▸), *DIAMOND* (Brandenburg, 2006 ▸) and *publCIF* (Westrip, 2010 ▸).

## supplementary crystallographic information

### Crystal data

**Table:**

C_14_H_14_N_4_O_2_·H_2_O	*F*(000) = 608
*M**_r_* = 288.31	*D*_x_ = 1.373 Mg m^−^^3^
Monoclinic, *P*2_1_/*n*	Cu *K*α radiation, λ = 1.54184 Å
*a* = 12.4784 (4) Å	Cell parameters from 5162 reflections
*b* = 5.0247 (1) Å	θ = 4.0–75.9°
*c* = 22.2410 (6) Å	µ = 0.82 mm^−^^1^
β = 90.170 (3)°	*T* = 100 K
*V* = 1394.51 (6) Å^3^	Prism, colourless
*Z* = 4	0.09 × 0.07 × 0.03 mm

### Data collection

**Table:**

XtaLAB Synergy Dualflex AtlasS2 diffractometer	2441 reflections with *I* > 2σ(*I*)
Detector resolution: 5.2558 pixels mm^-1^	*R*_int_ = 0.053
ω scans	θ_max_ = 76.7°, θ_min_ = 4.0°
Absorption correction: gaussian (Crysalis PRO; Rigaku OD, 2018)	*h* = −14→15
*T*_min_ = 0.921, *T*_max_ = 1.000	*k* = −6→6
16961 measured reflections	*l* = −27→28
2871 independent reflections	

### Refinement

**Table:**

Refinement on *F*^2^	Primary atom site location: structure-invariant direct methods
Least-squares matrix: full	Secondary atom site location: difference Fourier map
*R*[*F*^2^ > 2σ(*F*^2^)] = 0.043	Hydrogen site location: mixed
*wR*(*F*^2^) = 0.116	H atoms treated by a mixture of independent and constrained refinement
*S* = 1.04	*w* = 1/[σ^2^(*F*_o_^2^) + (0.0553*P*)^2^ + 0.7659*P*] where *P* = (*F*_o_^2^ + 2*F*_c_^2^)/3
2871 reflections	(Δ/σ)_max_ < 0.001
202 parameters	Δρ_max_ = 0.30 e Å^−^^3^
0 restraints	Δρ_min_ = −0.24 e Å^−^^3^

### Special details

**Table:**

Geometry. All esds (except the esd in the dihedral angle between two l.s. planes) are estimated using the full covariance matrix. The cell esds are taken into account individually in the estimation of esds in distances, angles and torsion angles; correlations between esds in cell parameters are only used when they are defined by crystal symmetry. An approximate (isotropic) treatment of cell esds is used for estimating esds involving l.s. planes.

### Fractional atomic coordinates and isotropic or equivalent isotropic displacement parameters (Å^2^)

**Table:**

	*x*	*y*	*z*	*U*_iso_*/*U*_eq_	
O1	0.39488 (9)	−0.1106 (2)	0.53440 (5)	0.0211 (3)	
O2	0.28502 (10)	0.4170 (2)	0.45056 (5)	0.0245 (3)	
N1	0.51280 (11)	0.8092 (3)	0.72982 (6)	0.0230 (3)	
N2	0.40224 (11)	0.3337 (3)	0.55256 (6)	0.0178 (3)	
H2N	0.3890 (16)	0.486 (4)	0.5378 (9)	0.021*	
N3	0.26914 (11)	−0.0297 (3)	0.43753 (6)	0.0176 (3)	
H3N	0.2848 (16)	−0.182 (4)	0.4529 (8)	0.021*	
N4	−0.08573 (13)	0.1419 (4)	0.36223 (9)	0.0434 (5)	
C1	0.52700 (13)	0.6284 (3)	0.68624 (7)	0.0205 (3)	
H1	0.598157	0.573079	0.677719	0.025*	
C2	0.44417 (12)	0.5164 (3)	0.65271 (7)	0.0176 (3)	
C3	0.34062 (13)	0.6008 (3)	0.66496 (7)	0.0202 (3)	
H3	0.281622	0.530908	0.642955	0.024*	
C4	0.32438 (13)	0.7884 (3)	0.70976 (7)	0.0224 (3)	
H4	0.254145	0.849326	0.718705	0.027*	
C5	0.41200 (13)	0.8860 (3)	0.74135 (7)	0.0227 (3)	
H5	0.400111	1.012319	0.772402	0.027*	
C6	0.47006 (13)	0.3104 (3)	0.60569 (7)	0.0202 (3)	
H6A	0.545984	0.329839	0.593668	0.024*	
H6B	0.461045	0.130847	0.623270	0.024*	
C7	0.37291 (12)	0.1213 (3)	0.52134 (7)	0.0163 (3)	
C8	0.30359 (12)	0.1859 (3)	0.46578 (7)	0.0170 (3)	
C9	0.20743 (13)	−0.0186 (3)	0.38182 (7)	0.0196 (3)	
H9A	0.228818	−0.169871	0.355973	0.024*	
H9B	0.225732	0.147561	0.360270	0.024*	
C10	0.08770 (13)	−0.0283 (3)	0.39089 (7)	0.0199 (3)	
C11	0.02169 (15)	0.1432 (4)	0.35990 (9)	0.0370 (5)	
H11	0.054734	0.272529	0.334924	0.044*	
C12	−0.13026 (14)	−0.0379 (4)	0.39790 (8)	0.0304 (4)	
H12	−0.206194	−0.042293	0.400717	0.036*	
C13	−0.07261 (17)	−0.2165 (5)	0.43067 (11)	0.0486 (6)	
H13	−0.107827	−0.342052	0.455703	0.058*	
C14	0.03821 (16)	−0.2127 (5)	0.42700 (10)	0.0450 (6)	
H14	0.079722	−0.336857	0.449325	0.054*	
O1W	0.71328 (9)	0.9787 (2)	0.77119 (5)	0.0217 (3)	
H1W	0.642 (2)	0.942 (4)	0.7593 (9)	0.033*	
H2W	0.7244 (18)	1.141 (5)	0.7574 (10)	0.033*	

### Atomic displacement parameters (Å^2^)

**Table:**

	*U*^11^	*U*^22^	*U*^33^	*U*^12^	*U*^13^	*U*^23^
O1	0.0241 (6)	0.0137 (5)	0.0254 (6)	0.0006 (4)	−0.0029 (4)	0.0011 (4)
O2	0.0323 (7)	0.0139 (5)	0.0272 (6)	0.0018 (5)	−0.0065 (5)	0.0007 (4)
N1	0.0189 (7)	0.0242 (7)	0.0261 (7)	−0.0010 (5)	−0.0026 (5)	−0.0033 (5)
N2	0.0207 (7)	0.0122 (6)	0.0206 (6)	0.0009 (5)	−0.0012 (5)	0.0013 (5)
N3	0.0192 (7)	0.0131 (6)	0.0205 (6)	0.0005 (5)	−0.0011 (5)	−0.0001 (5)
N4	0.0187 (8)	0.0514 (11)	0.0600 (11)	0.0000 (7)	−0.0009 (7)	0.0268 (9)
C1	0.0160 (7)	0.0205 (8)	0.0248 (8)	0.0001 (6)	−0.0022 (6)	−0.0008 (6)
C2	0.0178 (7)	0.0156 (7)	0.0195 (7)	−0.0013 (6)	−0.0013 (6)	0.0023 (5)
C3	0.0161 (7)	0.0219 (8)	0.0227 (7)	−0.0035 (6)	−0.0016 (6)	0.0002 (6)
C4	0.0170 (8)	0.0263 (8)	0.0239 (7)	−0.0002 (6)	0.0023 (6)	−0.0018 (6)
C5	0.0213 (8)	0.0243 (8)	0.0226 (7)	−0.0008 (6)	−0.0003 (6)	−0.0034 (6)
C6	0.0192 (8)	0.0175 (7)	0.0239 (7)	0.0014 (6)	−0.0037 (6)	−0.0018 (6)
C7	0.0151 (7)	0.0140 (7)	0.0198 (7)	−0.0001 (5)	0.0032 (6)	0.0010 (5)
C8	0.0168 (7)	0.0151 (7)	0.0192 (7)	0.0013 (6)	0.0028 (6)	0.0003 (5)
C9	0.0195 (8)	0.0196 (7)	0.0197 (7)	−0.0003 (6)	−0.0004 (6)	−0.0012 (6)
C10	0.0206 (8)	0.0193 (7)	0.0198 (7)	−0.0013 (6)	0.0000 (6)	−0.0023 (6)
C11	0.0198 (9)	0.0432 (11)	0.0481 (11)	−0.0018 (8)	−0.0005 (8)	0.0262 (9)
C12	0.0187 (8)	0.0370 (10)	0.0355 (9)	−0.0035 (7)	0.0031 (7)	0.0028 (8)
C13	0.0268 (10)	0.0584 (14)	0.0605 (14)	−0.0057 (10)	0.0068 (9)	0.0343 (12)
C14	0.0241 (10)	0.0509 (13)	0.0601 (13)	0.0014 (9)	−0.0001 (9)	0.0345 (11)
O1W	0.0186 (6)	0.0205 (6)	0.0261 (6)	−0.0009 (5)	−0.0027 (4)	0.0012 (5)

### Geometric parameters (Å, º)

**Table:**

O1—C7	1.2313 (18)	C4—H4	0.9500
O2—C8	1.2314 (18)	C5—H5	0.9500
N1—C5	1.341 (2)	C6—H6A	0.9900
N1—C1	1.341 (2)	C6—H6B	0.9900
N2—C7	1.3244 (19)	C7—C8	1.541 (2)
N2—C6	1.456 (2)	C9—C10	1.509 (2)
N2—H2N	0.85 (2)	C9—H9A	0.9900
N3—C8	1.323 (2)	C9—H9B	0.9900
N3—C9	1.4579 (19)	C10—C14	1.374 (2)
N3—H3N	0.86 (2)	C10—C11	1.376 (2)
N4—C12	1.326 (2)	C11—H11	0.9500
N4—C11	1.342 (2)	C12—C13	1.361 (3)
C1—C2	1.392 (2)	C12—H12	0.9500
C1—H1	0.9500	C13—C14	1.386 (3)
C2—C3	1.388 (2)	C13—H13	0.9500
C2—C6	1.507 (2)	C14—H14	0.9500
C3—C4	1.387 (2)	O1W—H1W	0.95 (2)
C3—H3	0.9500	O1W—H2W	0.88 (2)
C4—C5	1.387 (2)		
			
C5—N1—C1	117.34 (14)	O1—C7—N2	125.30 (14)
C7—N2—C6	121.32 (13)	O1—C7—C8	120.84 (13)
C7—N2—H2N	118.1 (13)	N2—C7—C8	113.84 (13)
C6—N2—H2N	119.8 (13)	O2—C8—N3	125.51 (14)
C8—N3—C9	122.84 (13)	O2—C8—C7	121.59 (13)
C8—N3—H3N	117.8 (13)	N3—C8—C7	112.89 (13)
C9—N3—H3N	119.4 (13)	N3—C9—C10	113.95 (12)
C12—N4—C11	116.55 (16)	N3—C9—H9A	108.8
N1—C1—C2	124.18 (15)	C10—C9—H9A	108.8
N1—C1—H1	117.9	N3—C9—H9B	108.8
C2—C1—H1	117.9	C10—C9—H9B	108.8
C3—C2—C1	117.51 (14)	H9A—C9—H9B	107.7
C3—C2—C6	123.21 (14)	C14—C10—C11	116.47 (16)
C1—C2—C6	119.28 (14)	C14—C10—C9	123.13 (15)
C2—C3—C4	119.13 (14)	C11—C10—C9	120.31 (15)
C2—C3—H3	120.4	N4—C11—C10	125.07 (17)
C4—C3—H3	120.4	N4—C11—H11	117.5
C5—C4—C3	119.17 (15)	C10—C11—H11	117.5
C5—C4—H4	120.4	N4—C12—C13	123.24 (17)
C3—C4—H4	120.4	N4—C12—H12	118.4
N1—C5—C4	122.66 (15)	C13—C12—H12	118.4
N1—C5—H5	118.7	C12—C13—C14	119.05 (18)
C4—C5—H5	118.7	C12—C13—H13	120.5
N2—C6—C2	112.50 (12)	C14—C13—H13	120.5
N2—C6—H6A	109.1	C10—C14—C13	119.61 (18)
C2—C6—H6A	109.1	C10—C14—H14	120.2
N2—C6—H6B	109.1	C13—C14—H14	120.2
C2—C6—H6B	109.1	H1W—O1W—H2W	103.3 (19)
H6A—C6—H6B	107.8		
			
C5—N1—C1—C2	−0.1 (2)	O1—C7—C8—O2	−176.62 (15)
N1—C1—C2—C3	0.8 (2)	N2—C7—C8—O2	4.9 (2)
N1—C1—C2—C6	−178.75 (14)	O1—C7—C8—N3	2.8 (2)
C1—C2—C3—C4	−0.5 (2)	N2—C7—C8—N3	−175.72 (13)
C6—C2—C3—C4	178.98 (14)	C8—N3—C9—C10	−94.71 (17)
C2—C3—C4—C5	−0.3 (2)	N3—C9—C10—C14	−48.5 (2)
C1—N1—C5—C4	−0.8 (2)	N3—C9—C10—C11	135.08 (17)
C3—C4—C5—N1	1.0 (3)	C12—N4—C11—C10	0.8 (3)
C7—N2—C6—C2	−146.60 (14)	C14—C10—C11—N4	−0.4 (3)
C3—C2—C6—N2	37.4 (2)	C9—C10—C11—N4	176.3 (2)
C1—C2—C6—N2	−143.09 (14)	C11—N4—C12—C13	−0.6 (3)
C6—N2—C7—O1	3.0 (2)	N4—C12—C13—C14	0.0 (4)
C6—N2—C7—C8	−178.56 (13)	C11—C10—C14—C13	−0.3 (3)
C9—N3—C8—O2	3.0 (2)	C9—C10—C14—C13	−176.9 (2)
C9—N3—C8—C7	−176.35 (12)	C12—C13—C14—C10	0.5 (4)

### Hydrogen-bond geometry (Å, º)

**Table:**

*D*—H···*A*	*D*—H	H···*A*	*D*···*A*	*D*—H···*A*
N2—H2*N*···O2	0.85 (2)	2.36 (2)	2.7279 (18)	107.0 (16)
N3—H3*N*···O1	0.86 (2)	2.299 (19)	2.6924 (18)	108.0 (15)
O1*W*—H1*W*···N1	0.95 (2)	1.86 (2)	2.7958 (18)	169 (2)
O1*W*—H2*W*···O1*W*^i^	0.88 (2)	1.97 (2)	2.8364 (15)	166 (2)
N2—H2*N*···O1^ii^	0.85 (2)	2.03 (2)	2.8227 (18)	155.2 (18)
N3—H3*N*···O2^iii^	0.86 (2)	2.02 (2)	2.8022 (18)	151.6 (17)
C9—H9*A*···O1*W*^iv^	0.99	2.45	3.3772 (19)	156
C6—H6*B*···*Cg*1^iii^	0.99	2.74	3.7043 (16)	166

Symmetry codes: (i) −*x*+3/2, *y*+1/2, −*z*+3/2; (ii) *x*, *y*+1, *z*; (iii) *x*, *y*−1, *z*; (iv) *x*−1/2, −*y*+1/2, *z*−1/2.
